# 3D culture of alginate-hyaluronic acid hydrogel supports the stemness of human mesenchymal stem cells

**DOI:** 10.1038/s41598-024-54912-1

**Published:** 2024-02-23

**Authors:** Amorn Pangjantuk, Palakorn Kaokaen, Phongsakorn Kunhorm, Nipha Chaicharoenaudomrung, Parinya Noisa

**Affiliations:** Laboratory of Cell-Based Assays and Innovations, School of Biotechnology, Institute of Agricultural Technology, Suranaree University of Technology, 111 University Avenue, Nakhon Ratchasima, 30000 Thailand

**Keywords:** Alginate, Hyaluronic acid, 3D culture, Mesenchymal stem cells, Stemness, Cell biology, Mesenchymal stem cells

## Abstract

The three-dimensional (3D) cell culture system is being employed more frequently to investigate cell engineering and tissue repair due to its close mimicry of in vivo microenvironments. In this study, we developed natural biomaterials, including hyaluronic acid, alginate, and gelatin, to mimic the creation of a 3D human mesenchymal stem cell (hMSC) extracellular environment and selected hydrogels with high proliferation capacity for 3D MSC culture. Human mesenchymal stem cells were encapsulated within hydrogels, and an investigation was conducted into the effects on cell viability and proliferation, stemness properties, and telomere activity compared to the 2D monolayer culture. Hydrogel characterization, cell proliferation, Live/Dead cell viability assay, gene expression, telomere relative length, and MSC stemness-related proteins by immunofluorescence staining were examined. The results showed that 3D alginate-hyaluronic acid (AL-HA) hydrogels increased cell proliferation, and the cells were grown as cellular spheroids within hydrogels and presented a high survival rate of 77.36% during the culture period of 14 days. Furthermore, the 3D alginate-hyaluronic acid (AL-HA) hydrogels increased the expression of stemness-related genes (*OCT-4*, *NANOG*, *SOX2*, and *SIRT1*), tissue growth and development genes (*YAP* and *TAZ*), and cell proliferation gene (*Ki67*) after culture for 14 days. Moreover, the telomere activity of the 3D MSCs was enhanced, as indicated by the upregulation of the human telomerase reverse transcriptase gene (*hTERT*) and the relative telomere length (T/S ratio) compared to the 2D monolayer culture. Altogether, these data suggest that the 3D alginate-hyaluronic acid (AL-HA) hydrogels could serve as a promising material for maintaining stem cell properties and might be a suitable carrier for tissue engineering proposals.

## Introduction

Cell therapy using human mesenchymal stem cells (hMSCs) has indeed been extensively studied in the fields of tissue engineering and regenerative medicine^1^. MSCs have demonstrated therapeutic potential in the treatment of a wide range of disorders, including musculoskeletal deviations, osteoarthritis, and neurological disorders^2,3^. MSCs are a type of adult stem cell that can be isolated from various sources, such as bone marrow, umbilical cord blood, and adipose tissue^4^. MSCs exhibited the ability to self-renew and differentiate into various cell types, including osteocytes, chondrocytes, cardiomyocytes, and adipocytes^5^. MSCs have been shown to encourage tissue repair by reducing inflammation and angiogenesis^6,7^. Additionally, they can also secrete factors that enhance cell function and stimulate endogenous repair mechanisms^8^. Due to limited cell survival, poor cell engraftment, and a lack of site-specificity, the differences of MSC in 2D and 3D culture to cell delivery^9,10^. Therefore, it is imperative to develop methods and efficient cell culture systems to enhance cell survival and function by creating a specific environment that is as close as possible to in vivo context.

For cell culture investigations, 2D cell culture models have been adopted because of their simplicity and convenience^11^. Cells are grown as a monolayer on a flat surface, typically a plastic flask or dish. While 2D culture has been widely adopted, it has limitations when it comes to mimicking the complex cellular environment found in vivo^12^. Cell attachment occurs on only one side of the cell, and cell–cell interactions primarily happen at the perimeter of the monolayer, leading to a lack of cell complexity and limited cell–matrix interactions^13,14^. In contrast, 3D cell culture models provide a more physiologically relevant environment for cells by allowing them to grow in three dimensions, mimicking the spatial arrangement, cell–cell communication, and cell–matrix interactions found in vivo^15,16^. In a 3D culture system, cells are encapsulated within a hydrogel or scaffold that provides structural support and mimics the characteristics of the extracellular matrix (ECM)^17^. The hydrogel can be designed to have similar mechanical properties and biochemical cues as the native tissue ECM, enabling better cell–matrix interactions^18^. Thus, 3D cell culture is an alternative to cell studies and research because it is consistent and close to in vivo.

Hydrogels are commonly employed as scaffolds for cell therapy and tissue engineering because of their adaptability, biocompatibility, and ease of production^19,20^. Hydrogels with high hydrophilicity are biocompatible and can imitate natural tissues^21^. Hydrogels have been frequently used to create artificial ECM for in vitro studies of cellular function^22^. One of the most used biomaterials is alginate because of its biocompatibility, nontoxicity, and low immunogenicity, alginate has been used in a wide range of tissue engineering applications^23^. Alginate hydrogels increase the survival and function of MSCs^24^. Hyaluronic acid (HA) is a biomaterial with biological performance, porosity, degree of crosslinking, and compressive mechanical strength. It is commonly used in applications and designs to create 3D micro-environments in cells^25^. HA has a broad range of biological properties, such as angiogenesis, anti-inflammatory action, and chondrocyte differentiation^26,27^. And gelatin, which is formed by the hydrolysis of collagen protein. Gelatin biomaterials, which exhibit cross-linking and non-immunogenicity. These biomaterials enhanced the function of supporting cell attachment and cell retention in various cell types^28,29^. The alginate-gelatin hydrogels supported cell viability and spreading in fibroblasts^30^. However, previous studies have shown that alginate-gelatin hydrogels may be limited due to the viscosity and long-term stability of Ca2 + -crosslinked alginate hydrogels^31^. The choice of biomaterials as carriers for cell therapy strategies targeted at tissue regeneration has mostly been influenced by their extracellular matrix-like properties^32^. In the past, it was found that there were widespread studies of Alginate-hyaluronic acid hydrogel has been studied in delivery system, tissue engineering to evaluate the process to regulate the differentiation of MSCs, but AL-HA hydrogel for 3D culture has been studied in maintained of MSC cell conditions. There is still not enough information. Therefore, in our study, we are interested in applying 3D AL-HA hydrogel with low molecular weight to help promote MSC stability during proliferation before use in cell development or transplantation cells in the future. Therefore, alginate, hyaluronic acid, and gelatin are materials for the development of a 3D extracellular microenvironment for investigating the functions of cell survival and MSC stemness.

In this present study, we developed biomaterials, including hyaluronic acid, alginate, and gelatin, to mimic the creation of 3D hMSCs extracellular microenvironments and optimize cell proliferation in 3D hydrogel hMSC culture. Then, we selected the high proliferation of 3D hydrogel to investigate the proliferation capacity and survival of hMSCs in 3D hydrogels, which will be verified in comparison with the 2D monolayer culture. Finally, to verify whether 3D hydrogels can act as a potential effective way to maintain pluripotency and exhibit telomere activity, the expression of stemness-related genes, CD MSC-surface genes, tissue growth genes, cell proliferation gene, and telomere length activity on hMSCs in the 3D hydrogels will be evaluated compared to the 2D cell culture conditions.

## Materials and methods

### Chemicals and reagents

Hyaluronic acid (40,583) Low molecular weight (MW) 8000–15,000 Da, sodium alginate (A0682), gelatin (G1890), resazurin sodium salt (R7017), 4,6‐Diamidino‐2‐phenylindole (DAPI) (F6057), alizarin red solution, alcian blue 8GX solution and Oil Red O solution were purchased from Sigma‐Aldrich Chemical Co. (St. Louis, MO). 1X Alpha modified Eagle’s minimum essential medium (α-MEM) (AMEM) and L-glutamine was purchased from HyClone (HyClone, Logan, UT). Nonessential amino acids (NEAA), MEM α, nucleosides, no phenol red, Fetal bovine serum (FBS), 1X trypsin TrypLE™ Express 1X, and penicillin–streptomycin are purchased from Gibco (Gibco, CA, USA). Calcein‐AM and propidium iodide (PI) were purchased from Life Technologies Inc. (Carlsbad, CA). Antibodies anti-CD73, CD90, and CD105 were purchased from Merck (Merck KGaA, Darmstadt, Germany), and antibodies anti-phospho-SIRT1 were purchased from Affinity Biosciences (Cincinnati, USA).

### hMSC culture

The Umbilical cord-derived hMSCs (hUC-MSCs) was derived from I Wellness Co., Ltd. (Mueng, Nakhon Ratchasima, Thailand) by a healthy female donor aged 39 years who obtained written informed consent in accordance with the Human Research Ethics Committee under ethically acceptable terms of Suranaree University of Technology (EC63-06). Passage 1 cells, a homogeneous population of hMSCs were cultured in complete medium with α-MEM medium, 10% (v/v) FBS, 1% NEAA, 1% (v/v) L-glutamine, and 1% (v/v) penicillin–streptomycin. Cells were incubated at 37 °C in a humidified incubator atmosphere with 5% CO_2_. The media for 2D and 3D culture conditions were changed every 3 days. All experiments used cells from passage 3.

### Hydrogel preparation

Alginate, hyaluronic acid, and gelatin are dissolved in sterile deionized (DI) water under stirring at 37 °C to obtain a 1 wt% hydrogel aqueous solution and then sterilized with 0.20 µm. Hydrogels were seeded into 24-well plates, and the surface area of these hydrogels was irradiated with 365 nm UV light for 15 min (~ 1.7mWcm − 2, EA-180/FC, Spectroline, USA) to cause photo-crosslinking polymerization. These sterile hydrogels in 24-well plates were stored at − 20 °C for further use. Each sample was incubated at 37 °C to form hydrogels for 30 min. Three-weight-percent gels were used in all experiments.

### Cell seeding and 3D encapsulation of hMSCs in the hydrogels

hMSCs were seeded onto different biomaterial hydrogels for 2D and 3D encapsulation in a 24-well plate. 2D cell culture conditions were seeded 50,000 cells/well in medium. In 3D cell culture conditions, after hydrogels loading, The suspension of hMSCs were mixed with hydrogels at ratio 1:1 in the final concentration as 2 × 10^6^ cells/ml, following by Gwon et al.^33^. The 2D cell culture and 3D hydrogels were used to investigate the proliferation, cell survival and maintenance stemness of hMSCs.

### Scanning electron microscopy (SEM) image analysis

The structure and porosity of the hydrogels was observed using a SEM (SEM–EDS JEOL Model JSM-6010LV). After 1% alginate (AL), 1% hyaluronic acid (HA) with a low molecular weight preparation, the 3D AL-HA were prepared in ratio 1:1 and loaded into 96 well plates. This 3D AL-HA was mounted by using electrically silver conductive paints and coated with a thin layer of gold. Coated samples were scanned and photographed at an acceleration voltage of 3.0 kV The morphology of 3D AL-HA were observed under the SEM. Coated samples were photographed and examined the pore size (n = 5).

### Mechanical properties of the hydrogels

The mechanical properties of 3D hydrogels after freeze-drying were studied as described^34^ and determined by Texture profile analysis (TPA) methods using TA.XTplus100C Texture Analyzer (Texture Technologies Corp. and Stable Micro System, Ltd. Hamilton, MA). The samples were analyzed with two sequential compression tests with a 2 mm diameter cylinder probe. The TPA test parameters used were a pre-test speed of 5 mm/s, a test speed of 0.2 mm/sec, and a post-test speed of 5.0 mm/sec, and the strain rate was 50% for analyzing the hardness, adhesiveness, and Young’s modulus. Three repeated measurements for each of the hydrogels.

### Swelling ratio measurement

The 3D hydrogels (1:1 AL-HA) were incubated with PBS at 37 °C, weighed, and removed at the times specified. The swelling ratios were determined by comparing the weight of swollen gels (Ws) to freeze-dried gels (Wd) at 7 and 14 days, using the following formula: The swelling ratio (%) = Ws − Wd/Wd * 100%. Each measurement was carried out three times.

### The design biomaterial hydrogels by cell proliferation assay

The proliferation of hMSCs was measured by the resazurin assay, as previously described^34^. After seeding hMSCs according to the seeding steps above, both 2D cells and 3D hydrogels were cultured in 24-well plates. The media for 2D and 3D culture conditions were changed every 3 days and cultured for 15 days. The 3D biomaterial hydrogel includes 1% alginate (AL), 1% hyaluronic acid (HA) with a low molecular weight (MW) 8000–15,000 Da, 1% gelatin (GE), 1:1 AL-HA, 1:1 AL-GE, 1:1 HA-GE, and 1:1:1 AL-HA-GE. hMSCs were washed with PBS (phosphate buffered saline) and added the resazurin solution 25 µg resazurin/1 ml phenol red free α-MEM medium), which was incubated at 37 °C for 20 min. After being transferred to a 96-well plate, the relative fluorescence unit (RFU) of the resazurin is determined using a fluorescence microplate reader (Varioskan, Thermo Fisher Scientific, Waltham, MA) These samples were measured at an excitation wavelength of 530 nm, and an emission wavelength of 590 nm, and the average RFU was calculated after subtracting this average background signal from each of the wells (hydrogel without cells).

### Live/dead cell viability assay

The vitality of MSCs in the 3D HA-Al hydrogels was evaluated using live/dead cell fluorescent double staining. MSCs in the HA-Al hydrogels were incubated with calcein AM (2 µM) and propidium iodide (4 µM) diluted in serum-free medium for 20 min at room temperature after being cultured for 3, 7, and 14 days. The living cells exhibit green fluorescence (calcein AM), and the dead cells exhibit red fluorescence (propidium iodide). The live/dead images in the HA-Al hydrogels were observed under a fluorescence microscope (ZOE™ Fluorescent Cell Imager, Bio‐Rad Laboratories, Hercules, CA) and the percentage of live and dead cells measured using the ImageJ program (magnification of 100 ×). These data were calculated by Fluorescence intensity of the sample *100/Average Fluorescence intensity of the control (n = 3).

### RNA Extraction and qPCR Analysis

To examine the mRNA expression of hMSC stemness-related genes (SOX2, OCT4, NANOG, SIRT1, CD73, CD90, and CD105 genes), tissue growth and development genes (YAP and TAZ genes) and cell proliferation gene (Ki67 gene), in 2D and 3D culture conditions. After seeding hMSCs according to the seeding steps above, hMSCs were cultured for 3, 7, and 14 days. MSC spheroids were trypsinized and diluted by using stop solution. These cultured cells were harvested into 50 ml tube and centrifuged at 1500 rpm before determination of the mRNA expression. Total RNA from MSCs was extracted using a NucleoSpin RNA Plus kit (Macherey–Nagel, Dueren, Germany). A ReverTra Ace®qPCR RT Master Mix with gDNA Remover (Toyobo, Osaka, Japan) was utilized to convert the total mRNA into cDNA. The QuantStudio 5 Real-Time PCR System (Thermo Fisher Scientific) and qPCR BioSyGreen Mix Low‐Rox (PCR Biosystems, UK) were used to conduct qPCR analysis. The following cycling conditions by 40 cycles, 95 °C for 30 s, 60 °C for 30 s, and 72 °C for 45 s. The PCR products underwent melting curve analysis by being heated at 60 °C for 60 s and 95 °C for 15 s. The primer sequences are described in Table 1. Using the 2^−ΔΔCt^ method, relative mRNA expression was determined and normalized to the Ct of GAPDH (glyceraldehyde 3-phosphate dehydrogenase), a housekeeping gene, and the 2D culture condition.

**Table 1:** Primer sequences used for real‐time PCR.

Gene	Forward	Reverse
*SOX2*	5′-GCGAACCATCTCTGTGGTCT-3′	5′-GGAAATTTGGGATCGAACAA-3′
*OCT4*	5′-CAGTGCCCGAAACCCACAC-3′	5′-GGAGACCCAGCAGCCTCAAA-3′
*Nanog*	5′-TAATAACCTTGGCTGCCGTCTCTG-3′	5′-GCCTCCCAATCCCAAACAATACGA-3′
*Sirt1*	5´-GAATACCTCCACCTGAGTTG-3´	5´-GGCGAGCATAAATACCATCC-3´
*CD73*	5´-GCCTGGGAGCTTACGATTTTG-3´	5´- TAGTGCCCTGGTACTGGTCG -3´
*CD90*	5′-ATCGCTCTCCTGCTAACAGTCT -3′	5′-CTCGTACTGGATGGGTGAAC-3′
*CD105*	5′-TGCACTTGGCCTACAATTCCA-3′	5′AGCTGCCCACTCAAGGATCT-3′
*hTERT*	5′- GCCGATTGTGAACATGGACTACG-3′	5′-GCTCGTAGTTGAGCACGCTGAA-3′
*Ki67*	5′- CCTGTACGGCTAAAACATGGA-3′	5′-GCTGGCTCCTGTTCACGTA-3′
*YAP*	5′-CCTGCGTAGCCAGTTACCAA-3	5′-CCATCTCATCCACACTGT-3′
*TAZ*	5′-CTTGGATGTAGCCATGACCTT-3′	5′-TCAATCAAAACCAGGCAATG-3′
*Telomere*	5´-CGGTTTGTTTGGGTTTGGGTTTGGGTTTGGGTTTGGGTT-3´	5´-GGCTTGCCTTACCCTTACCCTTACCCTTACCCTTACCCT-3´
*36B4*	5´-GGTTACCTCCGAAACTGAAGA-3´	5´-CCTTTCATATGCAGTACATTAGCC-3´
*GAPDH*	5′-CTCTGCTCCTCCTGTTCGAC-3′	5′-TTAAAAGCAGCCCTGGTGAC-3′

### Telomere activity measurement by qPCR analysis

hMSCs were cultured in 2D and 3D culture conditions at 3, 7, and 14 days and determined human telomerase reverse transcriptase (hTERT) gene expression and telomere measurement analysis (T/S ratio). For the hTERT gene expression, total RNA was isolated by using a NucleoSpin RNA Plus kit (Macherey–Nagel, Dueren, Germany). RNA was converted into cDNA by using a ReverTra Ace®qPCR RT Master Mix with gDNA Remover (Toyobo, Osaka, Japan). The human telomerase reverse transcriptase (hTERT) gene is used to determine the regulation of telomerase activity and maintain telomere length, as previously described^35,36^. The qPCR BioSyGreen Mix Low‐Rox (PCR Biosystems, UK) and QuantStudio 5 Real-Time PCR System (Thermo Fisher Scientific) were used for all quantitative PCR reactions. The gene-specific primers used for qPCR are listed in Table 1. The mRNA expression was examined and normalized to GAPDH, a housekeeping gene, using the 2^−ΔΔCt^ method.

For the T/S ratio, DNA was extracted by using the genomic DNA extraction kit (Macherey–Nagel). The relative telomere length was measured using the qPCR amplification method, as previously described^37^. The relative telomere/single copy gene produced by this approach is inversely correlated with the mean telomere length. Except for the oligonucleotide primers, the components of the T and S PCR reactions were identical. The SYBR Green PCR master mix (PCR Biosystems, UK) was used to conduct all qPCR analysis. The telomere repeats (Tel PCR) were amplified in one PCR reaction, and the 36B4 gene, a single copy gene used as a control, was amplified in the second reaction using the QuantStudio 5 Real-Time PCR System (Thermo Fisher Scientific). The primer sequences of the Tel and 36B4 genes are shown in Table 1. The following cycling conditions, Tel PCR, 30 cycles of 95 °C for 15 s and 56 °C for 1 min, and 36B4 PCR, 30 cycles of 95 °C for 15 s and 56˚C for 20 s. The average duplicate telomere over the average duplicate single-copy gene served as the unit of measurement for relative telomere length. All the telomere lengths that were shown were relative to the 2D culture condition.

### Immunofluorescence staining

After seeding hMSCs according to the seeding steps above, hMSCs were cultured with 2D cells or 3D hydrogels in 24-well plates after being cultured for 7 days. The cells were fixed with 4% paraformaldehyde (PFA) for 30 min at room temperature, then washed three times with PBS. MSCs were incubated with blocking buffer solution (3% BSA and 0.5% Triton X-100 diluted with PBS) for 1 h. at 4 °C. Cells were strained with a 1:500 dilution of anti-CD73, anti-CD90, anti-CD105, anti-SIRT1, and incubated at 4 °C overnight. The cells were washed with two times of washing buffer solution (0.5% Triton X-100 diluted with PBS) for 1 h. at 4 °C and incubated with secondary antibodies for 1 h. After that, cells were washed with washing buffer and strained with DAPI to observe the nucleus of the cell. The images were observed using a fluorescence microscope (ZOETM Fluorescent Cell Imager, BIO-RAD).

### Flow cytometric method

The hMSC samples were washed with 2 ml staining Buffer and then centrifuged at 300 g for 5 min before transfer the cell sample sediment (1 × 106 cells in 100 µl) into a 5 ml flow cytometry tube. Then, 5 µl of each antibody positive markers (Mouse IgG1 Anti-Human CD73-PeCY7, Mouse IgG2A Anti-Human CD90-FITC and Mouse IgG1 Anti-Human CD105-PE) and negative Markers (Mouse IgG1 Anti-Human CD34-PE and Mouse IgG1 Anti-Human CD45-PC5) were added into the cell samples. These cells were incubated for 30 min at room temperature and in the dark. After the incubation, washed the cells with staining buffer and then centrifuge at 300 g for 5 min. Theses hMSC were dissolved with 200–400 µl of staining buffer and determined by using Cytomics FC500 Flow Cytometer at the laser wavelength range of Excited 488 nm and Emission 633 nm.

### hMSCs differentiation

For osteogenic differentiation, hMSCs were cultured for 21 days in α-MEM for 30 min at 25 °C. Differentiated cells were fixed in 4% formaldehyde for 30 min at 25 °C. The cells were stained with a 2% Alizarin Red solution for 20 min at 25 °C. To induce adaptogenic differentiation, hMSCs were cultured for 14 days in α-MEM. Following a 30-min fixation in 4% formaldehyde at 25 °C, differentiated cells were stained for 20 min at 25 °C using a 0.5% Oil Red O solution, and the chondrogenic differentiation, hMSCs were cultured for 21 days. and chondrogenic differentiation, MSCs were cultured for 21 days. Chondrocytes were fixed in 4% formaldehyde for 1 h at 25 °C and stained with Alcian Blue 8GX solution for 20 min at 25 °C. Three duplicate samples were used for the differentiation experiment.

### Statistical analysis

SPSS (version 16.0, SPSS Inc., USA) was used to conduct the statistical analysis. The data is displayed as mean SD values. Utilizing one-way ANOVA, multiple quantitative data sets were compared. The student’s t-test was used to compare the two quantitative data sets. The level required for statistical significance was *p* < 0.05.

## Results

### The design biomaterial hydrogel for 3D culture hMSCs

We developed the biomaterial hydrogels for 3D native ECM components in hMSCs by photopolymerization.First, we designed biomaterial hydrogels, including AL, HA, and GE, for optimizing cell proliferation in 3D hydrogel hMSC cultures. Then, we selected the high proliferation of 3D hydrogel to investigate the morphology and proliferation capacity in comparison to 2D cell cultures.

**Figure 1:**
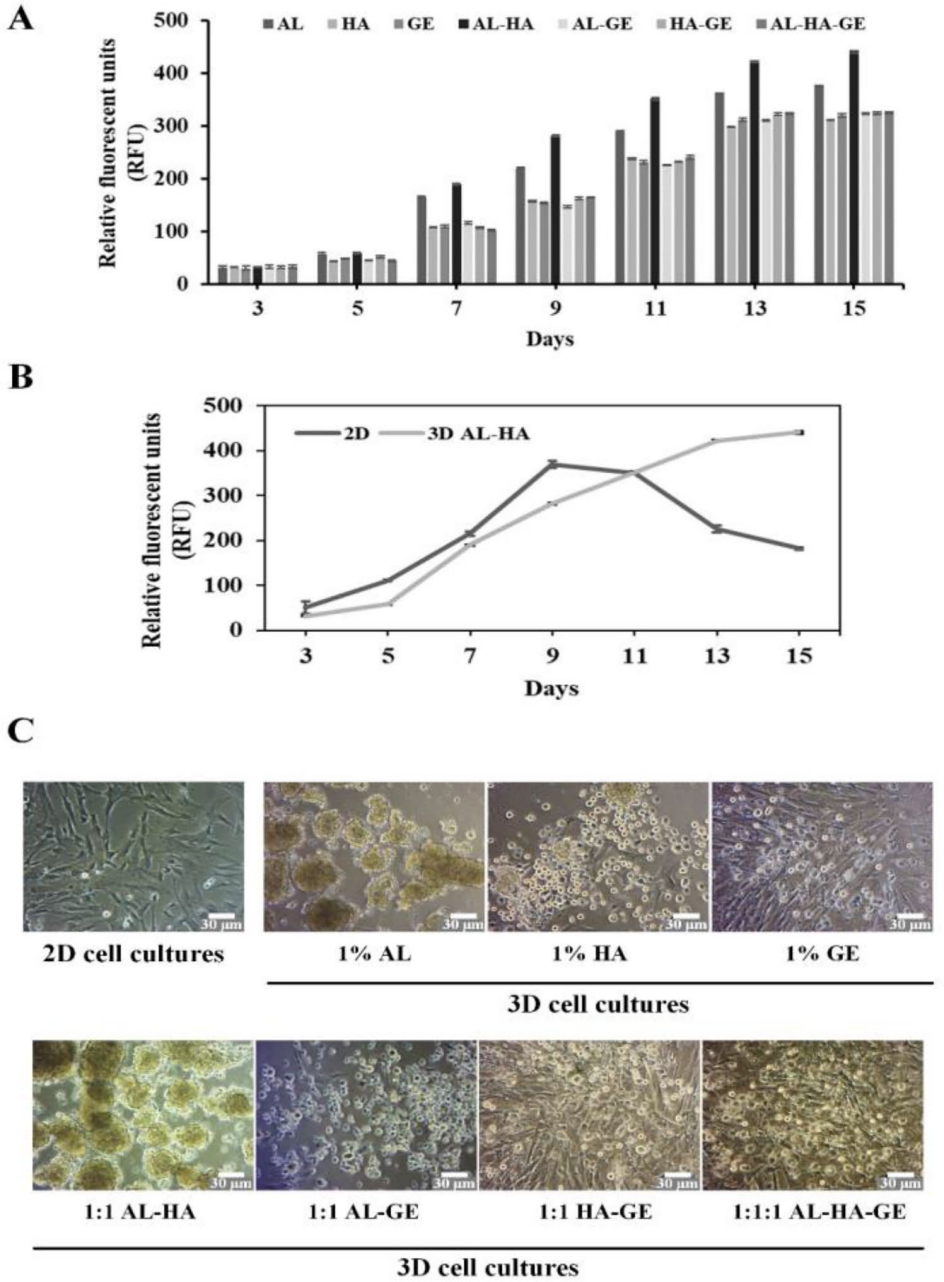
The cell proliferation and morphology of 3D hMSCs in biomaterial hydrogels. (**A**) The proliferation capacity of MSCs in various biomaterial hydrogel cultures for 15 days, as determined by the resazurin assay. (**B**) The proliferation rate of 2D and 3D AL-HA hydrogels during 15 days of culture and (**C**) The morphology of MSCs in 2D and 3D biomaterial hydrogels at day 7 of culture.

In this research, we design the biomaterial hydrogels (1% AL, 1% HA, 1% GE, 1:1 AL-HA, 1:1 AL-GE, 1:1 HA-GE, and 1:1:1 AL-HA-GE hydrogels) to be used for 3D hMSC cultures. Cell proliferation of biomaterial hydrogels was assessed by the resazurin assay, which measured the relative fluorescence unit (RFU). The results indicated that the proliferation rate of all hydrogel treatments increased with increasing culture time (days). However, after being cultivated for 15 days, it was discovered that the AL-HA hydrogels had a higher proliferative capability than the various biomaterial hydrogels (Fig. 1A). Next, we compared the proliferation rates of 2D cell culture and 3D AL-HA hydrogels, cultivated for 15 days. The results showed that during the early stages of culture, the 2D cell conditions grew faster than the 3D AL-HA hydrogels during the 9-day culture period. And then, the proliferation rate of 2D cell conditions decreased. However, after 10 days of continuous culture, the proliferation capacity of 3D AL-HA hydrogels was rapidly increasing (Fig. 1B). The population doubling time (PDT) in 5, 7 and 9 days during culture. The 2D cell culture were 2.1 ± 0.70, 4.09 ± 2.14, and 7.02 ± 5.67 days, and 3D AL-HA hydrogels were 1.12 ± 1.13, 3.62 ± 1.00, and 5.35 ± 1.70 days, respectively (data not shown). The morphology of hMSCs in the 2D monolayer cultures displays them as spindle-shaped flat cells, which is different from the cells in the 3D AL-HA hydrogels, which grew as cellular spheroids (The average size of hMSCs spheres approximately 25–62 µm) after culture for 7 days (Fig. 1C). These results indicate that 3D AL-HA hydrogels support cell proliferation and the continuous culture of hMSCs.

Our results with the AL-HA hydrogel showed a high proliferation capacity (RFU) compared with the various biomaterial hydrogels. Thus, we create a 3D mimic microenvironment in hMSCs using the AL-HA hydrogel to study cell survival and stemness maintenance, compared to 2D cell conditions.

### The characterization of hydrogels

**Figure 2:**
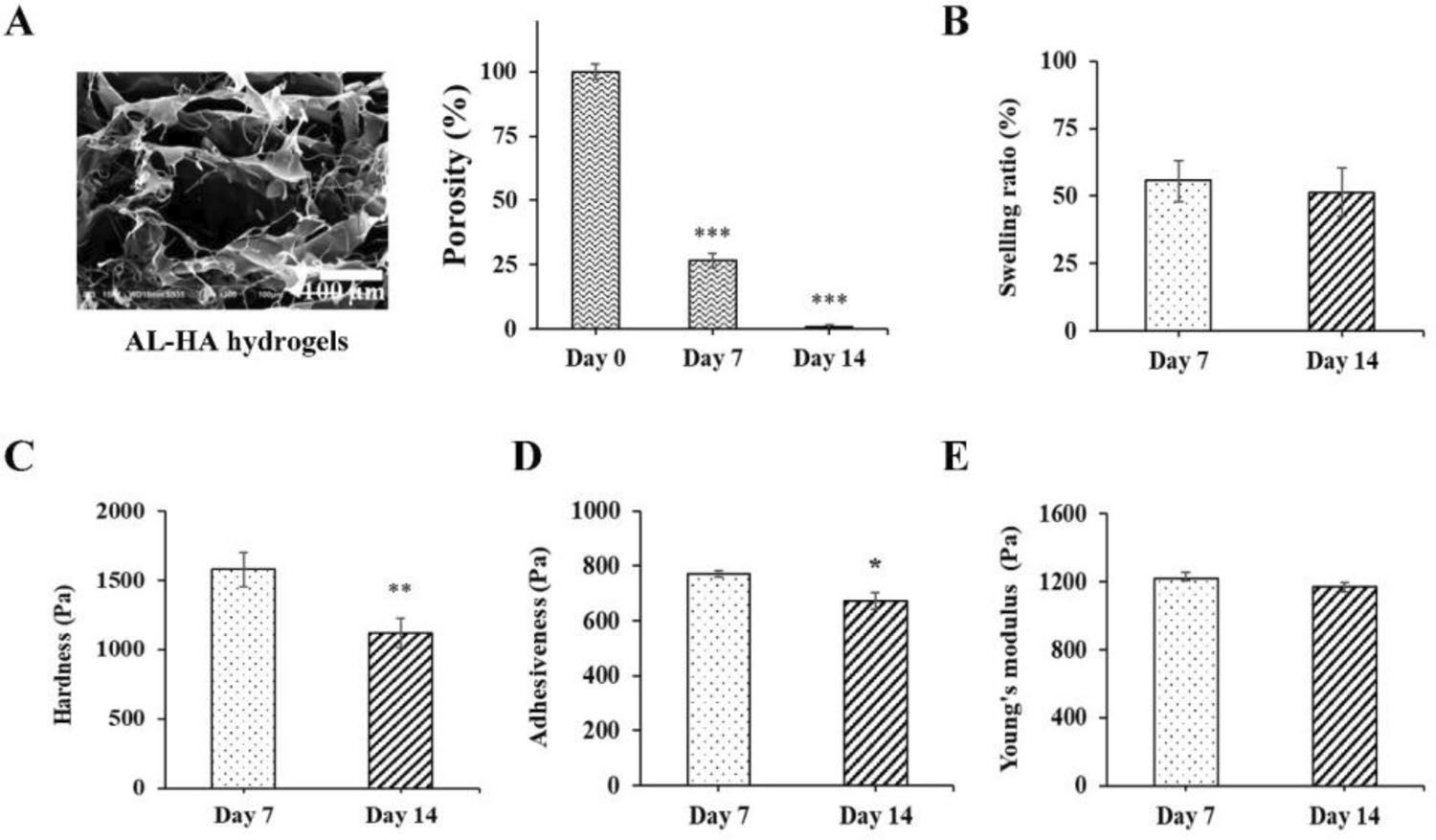
Characterization of AL-HA hydrogels. (**A**) SEM image and porosity of AL-HA hydrogels demonstrating the hydrogel’s microporous structure. (**B**) the swelling ratio of AL-HA hydrogels. Mechanical properties of AL-HA hydrogels (**C**–**E**) The hardness, adhesiveness, and Young’s modulus of AL-HA hydrogels, measured by Texture profile analysis (TPA) methods after 7 and 14 days. The values were presented as mean ± SD, n = 3 (*p < 0.05 and **p < 0.01 versus control).

We developed and examined the influence of AL-HA on the morphological structure, porosity, swelling ratio, and mechanical characteristics of hydrogels. Images obtained by scanning electron microscopy (SEM) were used to examine the morphological structure of the AL-HA hydrogels. The data suggested that the hydrogel matrix in AL-HA had a microporous structure. The pore size of this AL-HA having an average size of approximately 35–265 µm. The porosity of the AL-HA hydrogels on days 0, 7, and 14 was 100%, 26.72%, and 0.84%, respectively (Fig. 2A). As for the swelling ratio, the water absorption of AL-HA hydrogels showed the swelling ratio at 56% and 51% of their initial weight after 7 and 14 days, respectively (Fig. 2B). The mechanical properties of AL-HA hydrogels were determined by Texture profile analysis (TPA) methods. The results show that the AL-HA treatment exhibited hardness (1578 ± 121.29 Pa), adhesiveness (771 ± 9.96 Pa), and Young’s modulus (1220 ± 37.22 Pa) after culture for 7 days. In addition, the AL-HA treatment significantly decreased the hardness (1117 ± 106.43 Pa), adhesiveness (673 ± 30.70 Pa) after culture for 14 days. However, the Young’s modulus was slightly decreased (1170 ± 28.32 Pa) after culture for 14 days, when compared to 7 days (Fig. 2C–E). The results confirmed that the AL-HA hydrogels exhibit porous morphology, increase the swelling ratio of their initial weight, confirm the effect of water absorption, and their mechanical properties.

### Live/dead of hMSC cells in the 3D alginate-hyaluronic acid (AL-HA) hydrogels.

**Figure 3:**
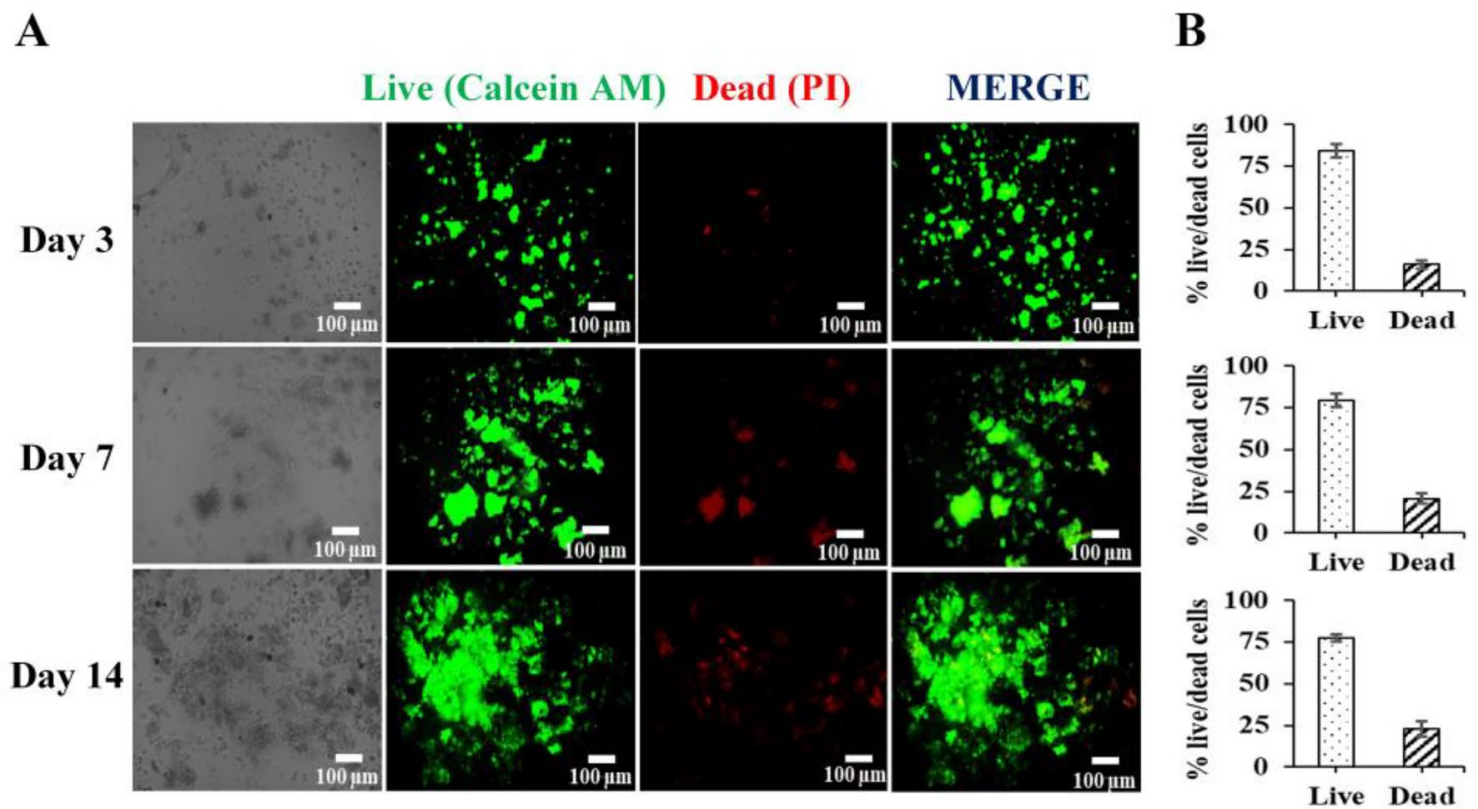
The viability of hMSCs in AL-HA hydrogels (**A**) fluorescence image of Live and dead cells. Living cells show green fluorescence (calcein AM), and dead cells emit red fluorescence (PI) and (**B**) The percentage of Live and dead cells after culture at 3, 7, and 14 days.

hMSCs were encapsulated in the AL-HA hydrogels (2 × 10^6^ cells/ml), cultivated for 3, 7, and 14 days (refer to the resazurin assay, which is in the lag phase, log phase and death phase, respectively). Live/Dead staining (calcein AM/PI) was used to determine the hMSCs viability in the AL-HA hydrogels. The results showed high cell viability of hMSCs after encapsulation in AL-HA hydrogels, most of the MSCs survived (green) and only a few dead cells (red) occurred during the culture period. The percentage of live cells was presented as 84.11 ± 3.71%, 79.26 ± 3.66%, and 77.36 ± 2.19% in the AL-HA hydrogels after being cultured at days 3, 7, and 14, respectively. Thus, 15.89 ± 2.11%, 20.74 ± 3.54%, and 22.64 ± 4.92% of the cells were dead at days 3, 7, and 14, respectively (Fig. 3A,B). Our results suggested that the AL-HA hydrogels may be a good strategy for supporting cell survival and 3D cell encapsulation of hMSCs.

### Effect of maintenance stemness, proliferation and telomere activity of hMSC cells in 3D alginate-hyaluronic acid (AL-HA) hydrogels

**Figure 4:**
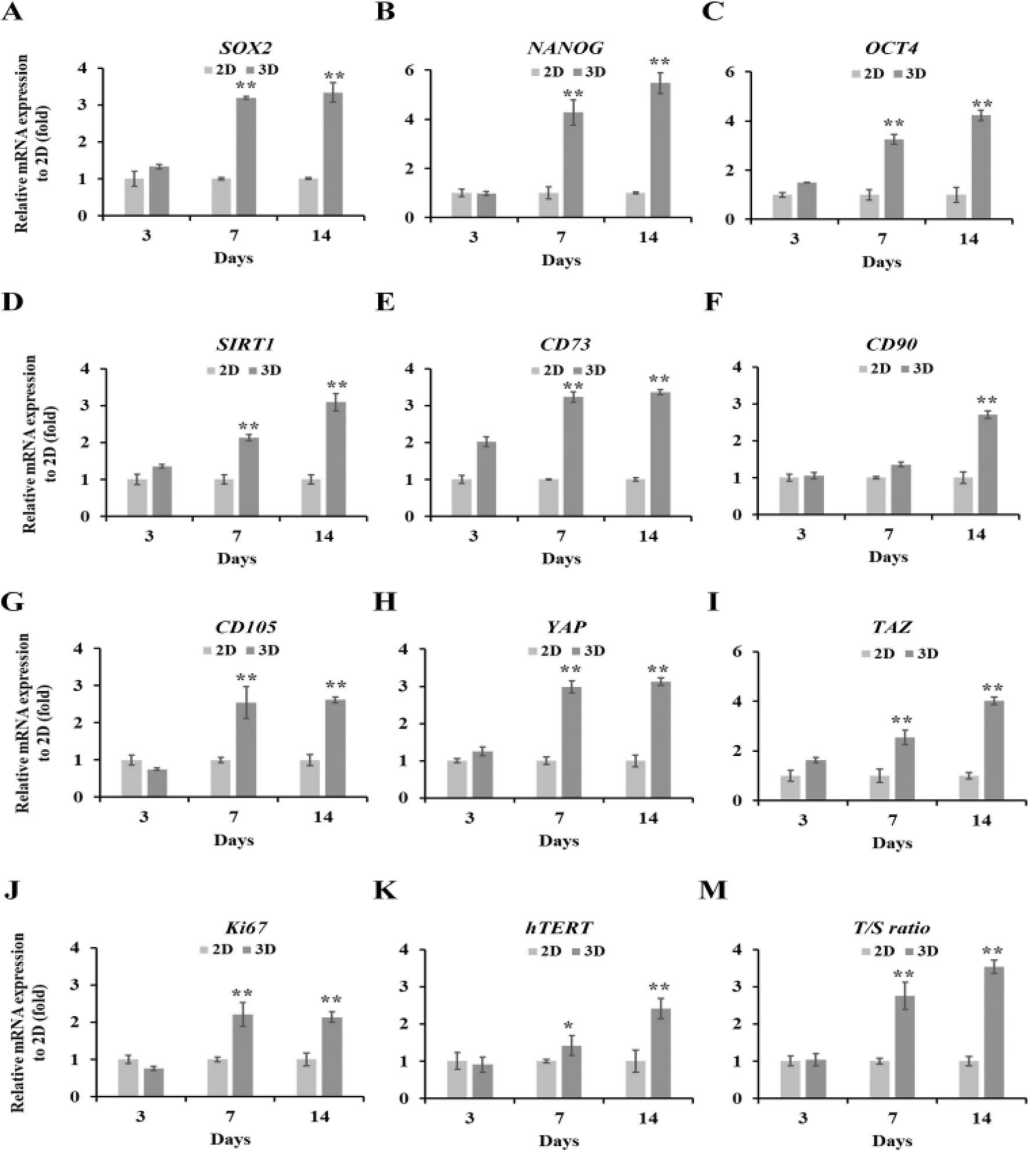
Effect of maintenance stemness, proliferation, and telomere activity on hMSC cells in 3D HA-Al hydrogels. The mRNA expression of MSC stemness-related genes, including (**A**) *SOX2*, (**B**) *NANOG*, (**C**) *OCT4*, and (**D**) *SIRT1*, the CD MSC-surface genes, including (**E** ) *CD73*, (**F**) *CD90*, and (**G**) *CD105*, the tissue growth genes, including (**H**) *YAP* and (**I**) *TAZ*, (**J**) the proliferation gene (*Ki67*), (**K**) the telomerase gene (*hTERT*), and (**M**) the relative telomere length (*T/S ratio*) for 3, 7, and 14 days, were examined by qPCR. GAPDH was used as a reference gene and normalized against the 2D culture condition. The values were presented as mean ± SD, n = 3 (*p < 0.05 and **p < 0.01 versus control).

To evaluate the expression of maintenance stemness, proliferation, and telomere measurement in the AL-HA hydrogel system of hMSCs, were assessed by qPCR using the GAPDH gene as a reference gene and compared to the 2D cell culture conditions. The stemness properties of hMSCs were determined by the MSC stemness-related genes (*SOX2*, *OCT4*, *NANOG*, and *SIRT1*) and CD MSC-surface genes (*CD73*, *CD90*, and *CD105*). Our findings showed that, after 14 days of cultivation, the stemness-related genes and CD MSC-surface genes were significantly upregulated in the AL-HA hydrogel by 3.3-, 4.3-, 5.4-, 3.1-,3.4-, 2.66-, and 2.68-folds, respectively, compared to 2D cell culture (Fig. 4A–G). Our results suggested that the 3D AL-HA hydrogels maintain the stemness of hMSCs.

The expression of tissue growth genes (*YAP* and *TAZ*) and cell proliferation gene (*Ki67*) was determined upon the Al-HA hydrogels of MSCs. The findings indicate that after day 14, the AL-HA hydrogel significantly increased the YAP, TAZ, and Ki67 genes by 3.1-, 4.0-, and 2.1-folds, respectively, in comparison to the 2D culture (Fig. 4H–J). These findings imply that the 3D AL-HA hydrogels enhanced the proliferation of hMSCs. To determine the telomere activity and T/S ratio in 2D and 3D Al-HA hydrogel systems. The results indicate that after day 14, the AL-HA hydrogel significantly increased the telomere activity (*hTERT)* and the relative telomere length (*T/S ratio*) by 2.4- and 3.5-folds, respectively, in comparison to the 2D culture (Fig. 4K, M). Our results suggested that the 3D AL-HA hydrogels increased the telomere activity of hMSCs. however, the stemness, tissue growth and development, cell proliferation and telomere activity related-genes at day 3 culture period in 2D compared to 3D AL-HA hydrogels were not significant mRNA expressions, While, day 7 culture period were significantly increased mRNA expressions (except CD90).

### Protein level of MSC stemness in the 3D alginate-hyaluronic acid (AL-HA) hydrogels

**Figure 5:**
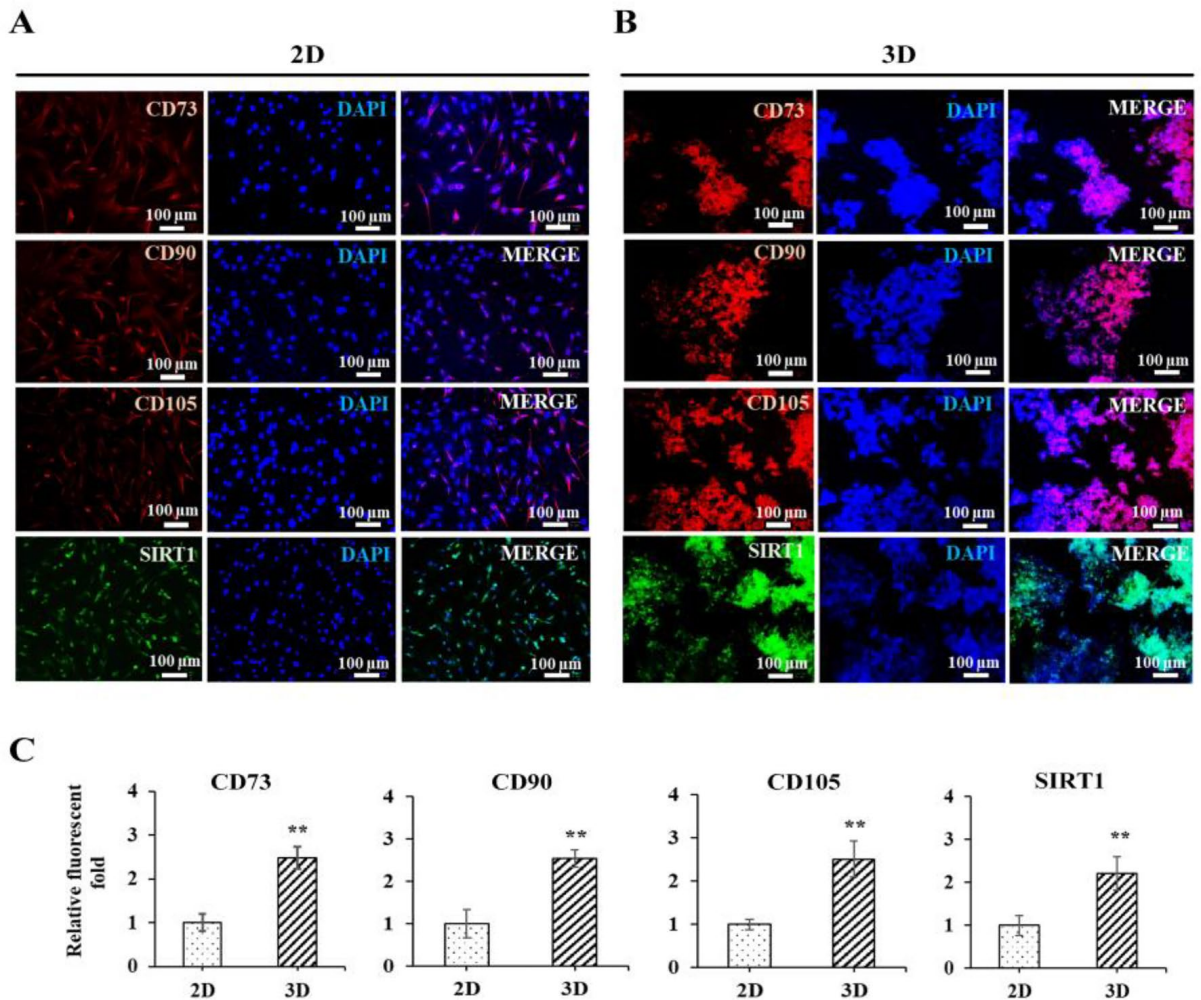
The protein level of MSC stemness-related proteins in 2D and 3D AL-HA hydrogels. Immunofluorescent images of MSC stemness-related proteins (CD73, CD90, CD105, and SIRT) in (**A**) 2D and (**B**) 3D AL-HA hydrogels at 7 days and (**C**) the fluorescence intensity image of MSC stemness-related proteins in 2D and 3D AL/HA hydrogels were quantified by the ImageJ Program. The values were presented as mean ± SD, n = 3 (*p < 0.05 and **p < 0.01 versus control).

Our findings show that 3D AL-HA hydrogels increased the mRNA expression of MSC stemness-related genes, maintaining the stemness of human mesenchymal stem cells. The level of MSC stemness-related proteins (CD73, CD90, CD105, and SIRT1 proteins) in the 3D AL-HA hydrogel of MSCs was therefore evaluated by immunofluorescent labeling and compared to the 2D cell culture conditions to further our investigation. The findings show that at day 7, compared to 2D cell culture conditions, the fluorescent images in the 3D AL-HA hydrogels significantly increased the fluorescence intensity of MSC stemness-related proteins (CD73, CD90, CD105, and SIRT1) (Fig. 5A–C). The overall results suggest that 3D AL-HA hydrogels enhanced the stemness properties of hMSCs.

### The characterization of hMSCs on the expression of MSCs surface markers and trilineage differentiation

**Figure 6:**
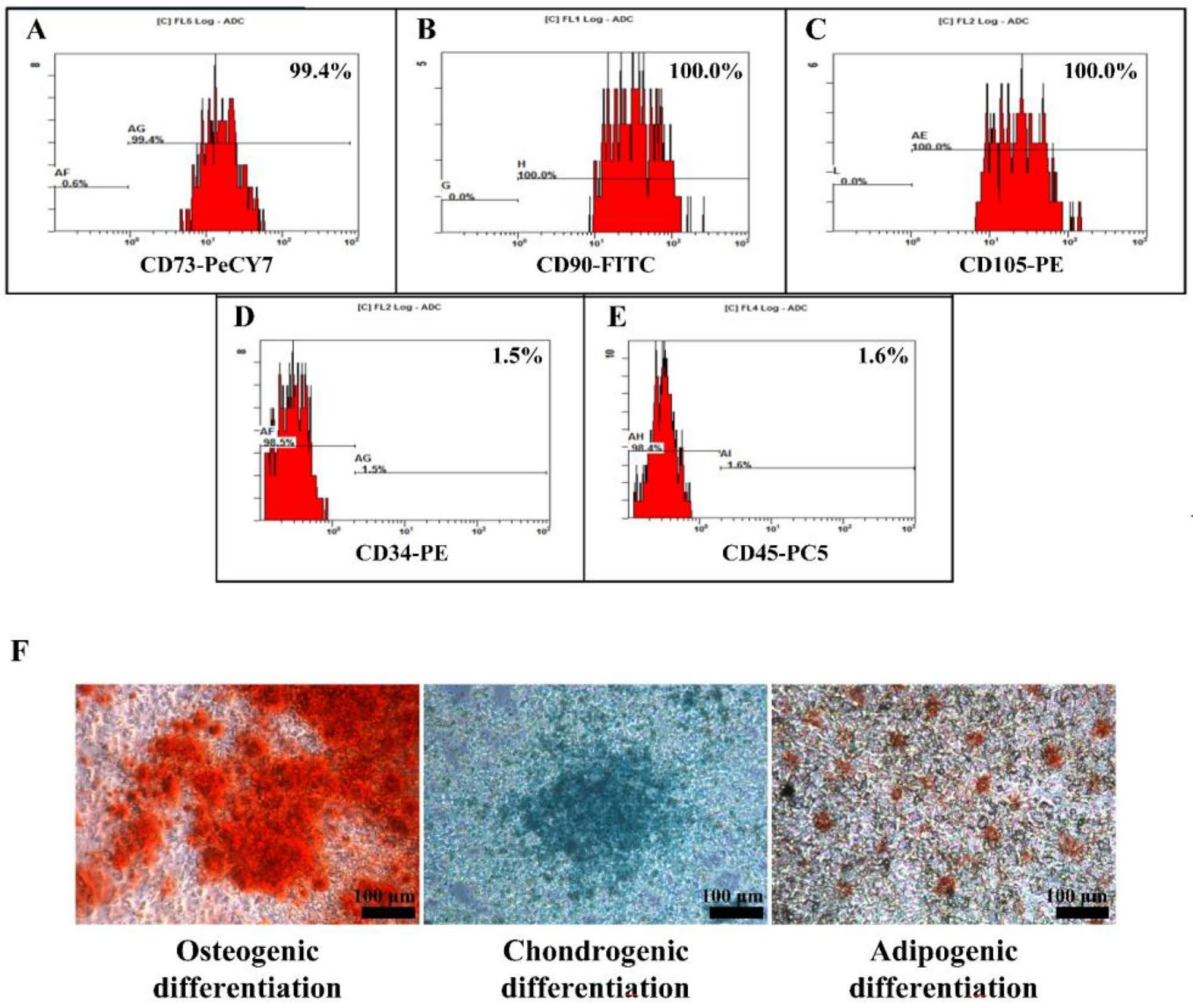
The characterization of hMSCs. The expression of MSCs surface markers. Representative flow cytometry analyses for the expression of the positive markers, (**A**) Mouse IgG1 Anti-Human CD73-PeCY7, (**B**) Mouse IgG2A Anti-Human CD90-FITC and (**C**) Mouse IgG1 Anti-Human CD105-PE, the negative Markers, (**D**) Mouse IgG1 Anti-Human CD34-PE and (**E**) Mouse IgG1 Anti-Human CD45-PC5. (**F**) Trilineage differentiation of hMSCs: osteogenic, chondrogenic, and adipogenic differentiation.

These hMSCs were characterized using positive markers (CD73, CD90, and CD105) and negative markers (CD34 and CD45) by using a flow cytometer, as shown in Fig. 6A–E. The results show that the positive markers (Mouse IgG1 Anti-Human CD73-PeCY7, Mouse IgG2A Anti-Human CD90-FITC, and Mouse IgG1 Anti-Human CD105-PE) exhibited the %Positive marker as 99.4%, 100.0%, and 100%, respectively. However, the negative markers (Mouse IgG1 Anti-Human CD34-PE and Mouse IgG1 Anti-Human CD45-PC5) show the positive marker only at 1.5% and 1.6%, respectively. The results suggest these hMSC surface markers have enabled the identification of hMSC.

The trilineage differentiation of hMSCs: osteogenic, chondrogenic, and adipogenic differentiation. The results showed that osteogenic differentiation exhibits calcium salt deposition on the cell surface, which forms calcium nodules, indicating the successful induction of hMSCs into osteoblasts. The adipogenic differentiation showed that lipid droplets were detected, a characteristic feature of adipogenic differentiation. Confirmation was done using red oil staining, a common method to visualize lipid droplets within cells, and the chondrogenic differentiation showed that proteoglycan synthesis was observed, as visualized by Alcian blue staining used to identify chondrogenic differentiation (Fig. 6F). These findings confirmed the characterization of cells as hMSCs and show the potential of hMSCs to differentiate into lineages of osteogenic, chondrogenic, and adipogenic differentiation.

## Discussion

Previous studies have found that the high molecular weight of 1 million Da of HA has lower solubility due to its highly hygroscopic and viscous properties, and that HA with a molecular weight of more than 200 kDa can result in cell proliferation rates that are reduced by up to 20%. While low molecular weight HA less than 50 kDa was found to increase the solubility of HA with lower viscosity and showed 90% or higher viability of hMSCs^38^. Additionally, a high M/G ratio of AL results in high flexibility, while a low M/G ratio results in a brittle gel. However, the main drawback of gelatin-based hydrogels is their poor mechanical stability and durability, which severely limits their broad application^39^. In this research, we develop the biomaterial hydrogels by photopolymerization for the 3D microenvironment of hMSCs using the natural biopolymers (alginate, hyaluronic acid, and gelatin) to support cell survival, maintain pluripotency and increase telomere activity of hMSCs. Our results designed and examined the proliferation of 3D biomaterial hydrogels (1% AL, 1% HA, 1% GE, 1:1 AL-HA, 1:1 AL-GE, 1:1 HA-GE, and 1:1:1 AL-HA-GE hydrogels) in MSCs. The results showed that the AL-HA hydrogels had a higher proliferation capability than the various biomaterial hydrogels. However, the proliferation capacity of another biomaterial increased with culture time, but the results of RFU were less than those of the AL-HA hydrogels, after being cultivated for 15 days. Another reason for this decision was the proliferation capacity of hMSC in various 3D hydrogels. When incubated with cell cultures for 13 to 15 periods of the other biomaterials in this study (except AL-HA at a ratio of 1:1), it was found that the growth was slight compared to AL-HA, which may be used for prolonged culture. The proliferation capacity of 3D AL-HA hydrogels, compared to the 2D cell culture, results showed that the 2D cell conditions grew faster than the 3D AL-HA hydrogels during the 11-day culture period, after that, the 2D cells grew steadily decreasing due to space limitation (disk, well), resulting in reduced cell–cell communication and cell-ECM interaction Thus, cell proliferation and cell attachment decreased. However, for further cell growth, there must be subculture (passage cells) for cell expansion. Next, the proliferation capacity of 3D AL-HA hydrogels was rapidly increasing during 15 days of continuous culture, according to the morphology of hMSCs in the 3D AL-HA hydrogels, which grew as cellular spheroids for support continuously grew, cellular aggregates developed, and the proliferation of hMSCs The 3D hydrogel crosslinks spontaneously at body temperature (37 °C). Thus, the hydrogel can form at body temperature for cell regeneration and chondrogenic differentiation.

Due to their biocompatibility, flexibility, and high-water content, biopolymer hydrogels have been applied in cell-based experiments and can serve as cues for cell attachment and proliferation^40^. The hydrogel, a 3D cross-linked hydrophilic polymer, has been employed as a 3D scaffold for investigations in cell therapy and drug delivery because of its adaptability, biocompatibility, non-immunogenicity, and ability to maintain shape^41^. Alginate is a natural biomaterial derived from brown sea algae for regenerative medicine and tissue remodeling due to its non-toxicity and simplicity of cross-linking^42^. The alginate hydrogels exhibit cell viability, migration, proangiogenic growth factor secretion, and bone regeneration in vitro^25^. The other natural hydrogel biomaterial that is a promising ECM for cell regeneration is hyaluronic acid (HA), which as the component in the extracellular matrix (ECM) of cartilage^43^. Previous data showed that the hyaluronic acid enhance cell adhesion, anti-inflammations and induce osteogenic differentiation^44^. Our data examined the design biomaterial hydrogel for 3D hMSCs by cell proliferation analysis, the result indicates that the AL-HA hydrogel showed the high cell proliferation than the other biomaterial hydrogels for continuous culture of hMSCs, according to the previous research, The 3D calcium (Ca)‐alginate scaffolds enhanced high proliferation capacity the growth rate increased gradually, continuous culture during 21 days in human glioblastoma cells^34^. These results suggested that the biomaterial of alginate (AL) and AL-HA support cell proliferation in various cell types. The next step was to investigate the hydrogels properties of AL-HA hydrogels and apply them to the 3D mimic extracellular matrix (ECM) of hMSCs, and then the survival and cell functions were examined and compared to 2D cell conditions.

The 3D biomaterials play a role in cell culture’s efficiency and achievement, they function effectively and can be used as a scaffold to support cell growth and differentiation^45^. Our results, we examined the AL-HA hydrogels on the Morphological characteristics by SEM image, results indicate that AL-HA showed the porous structure of the hydrogel matrix, according to the previous research, showed that the biomaterial hydrogels, including AL^46^, chitosan–gelatin^47^, and pullulane-collagen hydrogels also exhibits the porous morphology in the matrix of hydrogels^48^. The porous structure of the hydrogel matrix supports cell attachment, expansion, the ability to absorb water, the retention of substances in large quantities, and the exchange of nutrients, oxygens and waste with the cells while maintaining their shape^49^. Next, the swelling ratio measurement of AL-HA hydrogels was characterized, and our results confirm that the water absorption of AL-HA hydrogels (51% of their initial weight) remained constant for 14 days. Our data were consistent with the previous results of AL^50^, HA^51^, and 2-Aminoethyl methacrylate (AEMA)–modified hyaluronic acid (HA) hydrogels^52^. The swelling ratio is associated with water uptake and metabolite exchange, both of which are crucial for cell migration and proliferation^53^, and then the mechanical properties of AL-HA hydrogels were examined by Texture profile analysis (TPA) methods. These results confirmed that the mechanical properties of AL-HA hydrogel treatments decreased with culture time. The Young’s modulus in this study was closer to the chondrogenic tissue (~ 1 kPa)^54^. The hardness (stiffness) and Young’s modulus properties determine the structural integrity of the hydrogel and its ability to provide mechanical support to cells and tissues. Stiffer hydrogels can provide structural support for cell attachment and proliferation^55^.

Biomaterial hydrogels are used in cell treatment to create a 3D cell environment that enhances cell survival and functionality^56^. Thus, using live/dead fluorescence staining, we investigated hMSC survival in the 3D AL-HA hydrogels in this study. These investigations suggest that after 14 days of cultivation, this resulted in a high cell survival rate of 77.36%. Our findings demonstrate that the 3D AL-HA hydrogel promotes cell viability during hMSCs’ prolonged culture, according to the previous study of another biomaterial hydrogel that can exhibit high cell survival in various cell types, including the heparin-hyaluronic acid hydrogel, which showed a survival rate of human adipose-derived mesenchymal stem cells (ADSCs) above 95%^33^, the methacrylated collagen hydrogel, which showed a survival rate of 75% in the human osteosarcoma cell line^57^, and the alginate/polycaprolactone hydrogels, which showed high cell survival as 82% in chondrocytes^58^. The biomaterial hydrogels, especially the AL-HA in these studies, enhance cell survival. Consequently, they are highly suited for cell regeneration and biomedical applications in Human mesenchymal stem cells.

To study the effects of 3D AL-HA hydrogels on maintenance stemness, proliferation, and telomere activity in hMSC cells, they were measured by qPCR analysis and compared the results to those obtained from 2D cell culture. The stemness properties exhibited to the ability of stem cells to self-renew and differentiate into various cell types^59^. The stemness-related genes (*SOX2*, *OCT4*, *NANOG*, and *SIRT1*) are transcription factors that play important roles in maintaining the pluripotent state of embryonic stem cells^60^ . These genes have been found to be expressed in various stem cell types, including Bone marrow mesenchymal stem cells (BM-MSCs)^61^, and Adipose-derived stem cells (ASCs)^62^. Our studies have shown that stemness-related genes, including SOX2, OCT4, NANOG, and SIRT1 genes, were upregulated in 3D AL-HA hydrogels. Additionally, the expression of CD MSC surface genes, including CD73, CD90, and CD105 genes on MSCs was enhanced in these 3D hydrogels compared to traditional 2D cell culture. This suggests that the 3D AL-HA hydrogels may provide a more favorable environment for the maintenance of hMSCs. The transcriptional expression of Ki67 that is present in cells during active phases of the cell cycle. Therefore, measuring the transcriptional expression of Ki67 can provide information about the proliferation rate of cells^63^. Our results confirmed that the 3D AL-HA hydrogel significantly increased the Ki67 gene in comparison to the 2D culture. According to the proliferation capacity of this study, the data exhibited 3D AL-HA hydrogels that grew faster than the 2D culture after 11 days of long-term culture, the proliferation is necessary for the expansion of hMSC populations. Thus, these data imply that the AL-HA hydrogels provided a supportive environment for the growth and multiplication of 3D hMSCs. In addition, the 3D hMSCs in AL-HA were significantly higher expression levels of YAP and TAZ genes. Previous studies, showed that the 3D hydrogels increased the expression of YAP and TAZ genes, which are related to tissue growth, cell proliferation, and apoptosis in hMSCs and mMSCs^64–66^ Our results showed that 3D cell-substrate interaction of YAP/TAZ as a key regulator of mechanotransduction by translating extracellular matrix (ECM) has the interplay in cell–cell communication and enhances the YAP/TAZ expression proteins more than 2D cell cultures^67^. Next, to compare the telomere activity in 2D and 3D AL-HA hydrogels measured by the hTERT gene expression. In our study, the hTERT gene was significantly upregulated in the AL-HA hydrogel systems. The hTERT gene plays a crucial role in telomerase activity, and the expression of this gene is strictly controlled in many cell types to preserve genomic stability and avoid cellular senescence^68^. Previous studies have confirmed that, when combined with the biomaterial hydrogels, telomerase-immortalized human adipose-derived stem cells (hASC/hTERT) showed advantageous properties for cell proliferation and differentiation^69^. Apart from that, the relative telomere length (*T/S ratio*) in HA/Al hydrogels exhibited higher than the 2D cell culture in our studies. The standard method for calculating telomere length is the T/S ratio, which relates the number of telomeres repeats to that of a single-copy gene, such as 36B4^70^. The maintenance of the integrity of chromosomal ends during cell division depends on telomere activity^71^. According to our research, the utilization of 3D AL-HA hydrogels enhanced the telomere activity of hMSCs.

In our studies, findings indicate that 3D AL-HA hydrogels increased the mRNA expression of MSC stemness-related genes and maintained the stemness of hMSCs. We examined the level of MSC stemness-related proteins (CD73, CD90, CD105, and SIRT1) in the 3D hydrogel and compared it to 2D cell culture conditions. The results showed that the fluorescent images in the 3D AL-HA hydrogels demonstrated an enhanced fluorescence intensity of MSC stemness-related proteins compared to 2D cell culture. However, interestingly, when comparing MSC stemness-related CD90 surface protein expression between 2 and 3D AL-HA culture, it was found that on day 7 of 3D culture, CD90 surface protein expression was significantly higher, indicating that forming MSC spheres promotes better protein expression on the cell surface. According to the previous result, the PEG (Polyethylene glycol)-based 3D printed hydrogels that exhibit the MSC stemness-related proteins (CD73, CD90 and CD105) higher fluorescence image than current 2D culture systems in MSCs for promote regenerative activity of keratinocytes and fibroblasts^72^. Mesenchymal stem cells (MSCs) ability to regenerate tissue depends on the maintenance of their stemness, which is accomplished by increasing the expression of genes and proteins. Overall, results suggest that 3D AL-HA hydrogels improve the stemness properties of human mesenchymal stem cells. This discovery provides a potential method for preserving and improving the stemness of hMSCs in 3D culture systems, which may have significant implications for tissue engineering and regenerative medicine.

## Conclusion

In summary, this research showed the development of a 3D Human mesenchymal stem cell culture system using AL-HA acid hydrogels. Our data showed that this system mimicked the in vivo microenvironment, supported the proliferation capacity, and exhibited high survival in continuous cell culture of hMSCs. In the 3D AL-HA hydrogel systems, Mesenchymal stem cells enhanced stemness properties, tissue growth, and proliferation compared with the 2D culture system. This suggests that the 3D AL-HA culture environment provided a more favorable condition for the maintenance of hMSCs’ characteristics and cell proliferation. Finally, the 3D AL-HA hydrogels enhanced the telomere activity compared to the 2D cell culture conditions. This indicates that the 3D AL-HA culture environment facilitated telomere activity and thus potentially enhanced the regenerative potential of hMSCs. Our research suggests that 3D AL-HA hydrogels can be a promising carrier for stem cell-based therapy and a variety of tissue engineering applications.

## Data Availability

The data analyzed in this study is available from the corresponding author on reasonable request.
